# Genome-Wide Analysis of *AGC* Genes Related to Salt Stress in Soybeans (*Glycine max*)

**DOI:** 10.3390/ijms26062588

**Published:** 2025-03-13

**Authors:** Wenmin Liu, Shuichan Yang, Yi Chen, Sujun Ye, Wenmin Lin, Xiaoya Lin, Yang Tang, Baohui Liu

**Affiliations:** Guangdong Key Laboratory of Plant Adaptation and Molecular Design, Innovative Center of Molecular Genetics and Evolution, School of Life Sciences, Guangzhou University, Guangzhou 510006, China; wenminliu@e.gzhu.edu.cn (W.L.); yangshuichan@e.gzhu.edu.cn (S.Y.); moon_withs@163.com (Y.C.); sujunye@e.gzhu.edu.cn (S.Y.); 2014400067@e.gzhu.edu.cn (W.L.); xiaoyalin@gzhu.edu.cn (X.L.)

**Keywords:** soybean, AGC protein kinase, gene family, salt stress, genome-wide analysis

## Abstract

The AGC protein kinase family plays a crucial role in regulating plant growth, immunity, and cell death, as well as responses to abiotic stresses such as salt-induced stress, which impact plant development and productivity. While the functions of AGC kinases have been thoroughly studied in model plants such as *Arabidopsis thaliana*, their roles in soybeans (*Glycine max*) remain poorly understood. In this study, we identified 69 *AGC* kinase genes in soybeans, which are unevenly distributed across 19 chromosomes and classified into five subfamilies: PDK1, AGCVI, AGCVII, AGCVIII, and AGC (other). Each subfamily shares similar exon–intron structures and specific motifs. Gene duplication and selection pressure analyses revealed that the *GmAGC* gene family is primarily expanded through segmental or whole-genome duplication, with all genes undergoing purifying selection during evolution. Promoter analysis identified numerous *cis*-regulatory elements associated with light, hormonal, and abiotic stress responses, including salt stress. The gene expression analysis demonstrated tissue-specific patterns, with the highest expression levels found in roots (19.7%). Among the 54 *GmAGC* genes analyzed using RT-qPCR, significant changes in expression were observed in the roots and leaves treated with sodium chloride, with most genes showing increased expression. These results illustrate the critical role of the soybean *AGC* kinase gene family in regulating responses to salinity stress. Our findings suggest that targeting specific *GmAGC* genes may enhance soybean resistance to salt toxicity, offering valuable insights for future crop improvement strategies.

## 1. Introduction

Effectively sensing and responding to external environmental stimuli is a vital process for plants’ survival and adaptation. Protein kinases play a key role in integrating developmental and environmental signals into specific cellular responses, enabling plants to adapt to diverse environmental conditions such as salinity, drought, and cold stress [1,2]. These enzymes catalyze the transfer of the γ-phosphate group from adenosine triphosphate (ATP) to specific serine, threonine, or tyrosine residues on target proteins, thereby altering their activity and functionality [3]. The plant protein kinase superfamily is one of the largest and most diverse gene families in plants, and it includes several key groups: protein kinase A/protein kinase G/protein kinase C (AGC), calcium/calmodulin-dependent kinase (CAMK), calcium-dependent protein kinases (CDPKs), sucrose non-fermenting1-related protein kinases (SnRKs), mitogen-activated protein kinases (MAPKs), and receptor-like kinases (RLKs) [4]. The AGC kinase subfamily, a group of serine/threonine protein kinases, includes cAMP-dependent protein kinase A (PKA), cGMP-dependent protein kinase G (PKG), and phospholipid-dependent protein kinase C (PKC), all of which are widely distributed in eukaryotes [5]. AGC kinases are critical in transmitting cellular signals, which they achieve by phosphorylating target proteins, thus regulating key processes such as cell division, membrane dynamics, and cell polarity [6]. In plants, AGC kinases play a critical role in regulating auxin transport and localization, responding to light signals, and adapting to both biotic and abiotic stresses, highlighting their versatile functions in maintaining cellular homeostasis and facilitating stress adaptation [7,8,9,10]. It has been established that SnRK2 kinases, which are activated by abscisic acid (ABA), play a pivotal role in drought tolerance by regulating protective mechanisms such as stomatal closure, reduced transpiration, and the activation of drought-responsive genes [11]. Similarly, MAPKs and salt overly sensitive (SOS) pathway kinases are essential for salt stress adaptation, as they maintain ion homeostasis and facilitate stress responses [12]. AGC kinases, particularly those that are activated by lipid signals through pathways involving *Phosphoinositide-Dependent Kinase 1* (*PDK1*), are involved in various stress response pathways, including immune responses and growth regulation under abiotic stress [13].

To date, the importance of the AGC kinase family in stress tolerance has been investigated in several plant species such as *Arabidopsis thaliana*, wheat, rice, *Brassica rapa*, and cotton [14,15,16,17,18,19,20]. In *A. thaliana*, the AGC kinase family comprises 39 members that are categorized into five subfamilies: PDK1, AGCVI, AGCVII, AGCVIII, and AGC (other) [21]. The PDK1 subfamily includes two highly conserved members, *PDK1* and *PDK2*, which function as master regulators by integrating lipid signals and activating other AGC kinases [22]. While initial studies suggested mild developmental defects in *A. thaliana PDK1*/*PDK2* double mutants, recent CRISPR/Cas9 knockout lines revealed severe auxin-related developmental abnormalities, highlighting their critical roles in plant growth [23,24,25]. Similarly, knockout of multiple *NtPDK1* alleles in tobacco led to severe developmental defects [26]. In rice, the *OsPdk1* AGC kinase plays a key role in maintaining basal resistance to salt-induced stress by regulating phosphorylation cascades that activate plant defense mechanisms [19]. The AGCVI subfamily contains two *p70 ribosomal S6 Kinase* genes (*S6K*), *S6K1* and *S6K2*, which regulate cell growth, proliferation, and stress responses [27]. Expression analysis indicates that *AtS6K1* is induced by UV-B and oxidative and genotoxic stresses, while *AtS6K2* is predominantly expressed in roots under salt stress [14]. The AGCVII subfamily consists of eight *Nuclear Dbf2-related* (*NDR*) kinases, which are conserved across plants, animals, and yeast and are involved in cell division, patterning, and programmed cell death [28]. In *A. thaliana*, *NDR2*, *NDR4*, and *NDR5* regulate late-stage pollen development and germination, with mutants displaying abnormal callose deposition and reduced fertilization rates [29]. In wheat (*Triticum aestivum*), *TaAGC1* (*NDR* homolog) is involved in immunity against *Rhizoctonia cerealis* by modulating reactive oxygen species (ROS)-related and defense-associated genes [17]. The AGC (other) subfamily includes four incomplete root hair elongation (*IRE*) kinases, which regulate root hair development and other processes. For example, *AtIREH1* regulates root deflection angles and stabilizes microtubule networks in *A. thaliana*, while *MtIRE* promotes the formation of nitrogen-fixing nodules in *Medicago truncatula* [30,31]. In cucumber, *CsIREH1* phosphorylates DELLA proteins to prevent their overaccumulation, ensuring normal growth [32]. Additionally, *AtIRE1a* and *AtIRE1b* mediate plant immunity via the unfolded protein response pathway, with *IRE1a* predominantly involved in pathogen-induced PR protein secretion and *IRE1b* primarily responding to tunicamycin-triggered stress [15]. The AGCVIII subfamily, which is unique to plants, is the largest AGC group in soybeans, consisting of 23 members [33]. This subfamily can be further separated into four subgroups: AGC1, AGC2, AGC3, and AGC4, each with distinct roles [34]. The AGC1 subgroup regulates plant growth, development, and hormone signaling. For instance, the *AtAGC1–4* genes influence seed size by controlling cell proliferation and embryo development [35]. In *A. thaliana*, four AGC2 kinases, including *Oxidative Signal-inducible 1* (*OXI1*)/*AGC2-1*, *AGC2-2*, *Unicorn* (*UCN*)/*AGC2-3*, and *Unicorn-like* (*UCNL*)/*AGC2-4*, are involved in root growth, oxidative stress signaling, and pathogen defense [22,36]. AGC3 kinases, including *PINOID* (*PID*), *Wavy Root Growth 1* (*WAG1*), and *WAG2*, phosphorylate auxin efflux carriers (PIN proteins), regulating auxin transport and distribution, which are essential for plant development [37]. AGC4 kinases, including *PHOTOTROPIN1* (*PHOT1*) and *PHOT2*, mediate blue light signaling and photomorphogenesis [38]. In cotton, the gene expression of the genes *GhAGC2*, *GhAGC8*, *GhAGC9*, *GhAGC10*, *GhAGC22*, *GhAGC23*, and *GhAGC24* is elevated by salt stress, indicating the involvement of *AGCs* in cotton’s response to salinity [18]. In *B. rapa*, the gene expression of *BrAGC21*, *BrAGC33*, *BrAGC37*, *BrAGC41*, *BrAGC55*, and *BrAGC56* is downregulated under salt stress, while the gene expression of *BrAGC09*, *BrAGC19*, *BrAGC26*, and *BrAGC44* is upregulated, emphasizing their crucial and multiple roles in response to salt stress [20].

Soybean (*Glycine max*) is a globally significant food and oil crop and a major source of plant protein for both human consumption and animal feed [39]. Salt toxicity is a major abiotic stress that negatively impacts soybean growth and development, resulting in reduced yields and limiting cultivation in saline-prone regions. Therefore, enhancing soybeans’ salt tolerance is crucial for sustaining productivity and expanding cultivation into these challenging environments. However, the role of the *AGC* gene family in soybeans’ response to salinity stress remains poorly understood. To address this gap, we performed a genome-wide analysis of the *AGC* kinase gene family in *G. max*, focusing on its roles in sodium chloride stress. By identifying and functionally characterizing the *GmAGC* gene family, this study aims to clarify its contributions to salinity stress tolerance. These findings provide a foundation for developing strategies aimed at enhancing soybeans’ resilience to environmental stresses, particularly salinity, and support the breeding of soybean cultivars with improved salt tolerance.

## 2. Results

### 2.1. Sequence Identification and Retrieval

A total of 39 *AGC* family genes were retrieved from *A. thaliana* using the TAIR database (Table S1). These sequences were then used as queries to identify homologous genes in *G. max* (Williams 82) via the Phytozome database, resulting in the identification of 69 homologous genes for further analysis (Table 1). To ensure the accuracy of the dataset, all retrieved sequences were re-examined for the presence of the conserved AGC domain using HMMER tools (Table S2).

### 2.2. Sequence Alignment and Phylogeny

**Figure 1:**
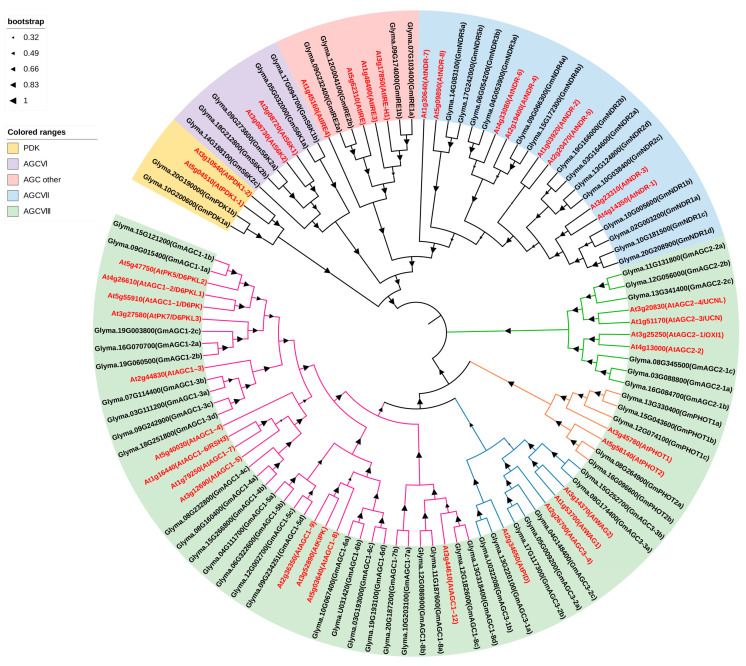
Phylogenetic tree of AGCs from *Glycine max* and *Arabidopsis thaliana*. Each colored range in the tree represents different subfamilies of AGC kinase. The four AGCVIII groups are represented by bootstrap lines of varying colors: an orange-yellow line for the AGC4 group, a purple line for the AGC1 group, a green line for the AGC2 group, and a blue line for the AGC3 group.

Phylogenetic analysis is an essential tool for elucidating the evolutionary relationships between genes. To investigate the AGC kinase gene family, a phylogenetic tree was constructed using protein sequences from *A. thaliana* and its homologs in *G. max*. *A. thaliana* was selected as the reference species due to its well-characterized status as a model plant, with a fully sequenced genome and classified *AGC* genes. This approach enabled the classification of soybean *AGC* genes based on clustering patterns observed in the phylogenetic tree. The analysis revealed that the 69 soybean *AGC* genes are grouped into five subfamilies (Figure 1). In accordance with established nomenclature, the soybean *AGC* genes were named based on their phylogenetic relationship and corresponding subfamilies (Table 1).

Within the PDK1 subfamily, two soybean genes, *GmPDK1a* and *GmPDK1b*, were identified as the closest homologs of the *A. thaliana PDK1* gene. Five genes homologous to the *A. thaliana S6K* gene were identified in the AGCVI subfamily: *GmS6K1a*, *GmS6K1b*, *GmS6K2a*, *GmS6K2b*, and *GmS6K2c*. Additionally, 14 soybean genes homologous to the *A. thaliana NDR* gene were classified into the AGCVII subfamily and designated as *GmNDR1a/b/c/d*, *GmNDR2 a/b/c/d*, *GmNDR3a/b*, *GmNDR4a/b*, and *GmNDR5a/b*. Four genes homologous to the *A. thaliana IRE* gene were assigned to the AGC (other) subfamily and named *GmIRE1a/b* and *GmIRE2a/b*. The remaining 44 genes were found in the AGCVIII subfamily, which was further classified into four groups: 26 genes in the AGC1 group, 6 in the AGC2 group, 7 in the AGC3 group, and 5 in the AGC4 group (Figure 1 and Table 1). The AGC2 and AGC4 (*GmPHOTs*) groups are located in the basal clade of AGCVIII. A phylogenetic analysis revealed that the *AGC* gene family has undergone significant expansion in *G. max*, particularly within the AGCVI, AGCVII, and AGCVIII subfamilies, where there were more members than in *A. thaliana*. These variations suggest that the soybean AGC kinase gene family has undergone different evolutionary paths or species-specific adaptations.

### 2.3. Physicochemical Properties of the GmAGC Kinase Family

The physicochemical properties of the GmAGC and AtAGC proteins were analyzed and are summarized in Table 1. The lengths of the GmAGC proteins ranged from 182 to 1302 amino acids, with molecular weights ranging from 19.99 kDa to 143.14 kDa. The isoelectric points (pI) of these proteins spanned from 5.41 to 9.71, with an average predicted pI of 7.41. Notably, certain proteins, including GmIREs, GmS6K2a, and GmPHOTs, exhibit higher molecular weights.

Subcellular localization predictions for the 69 GmAGC proteins, performed using the Softberry online tool, indicated that these proteins are distributed across various cellular compartments, including the nucleus, cytoplasm, plasma membrane, and extracellular spaces (Table 1). This diverse localization suggests that the GmAGC proteins play roles in a wide array of cellular processes.

### 2.4. GmAGC Localization on Chromosomes, Collinearity Analysis, Gene Duplication, and Ka/Ks Ratio

**Figure 2:**
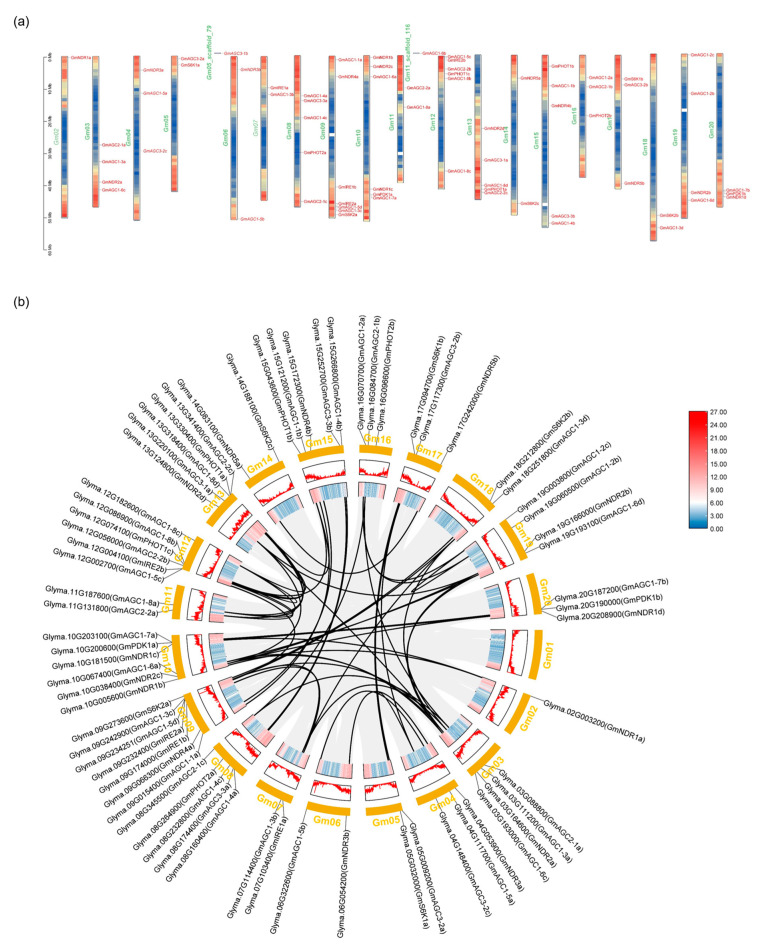
Chromosomal distribution and collinearity analysis of 69 *GmAGC* genes in *Glycine max*. (**a**) Chromosomal location of *GmAGCs* gene family members in *G. max*. The red to blue gradient represents varying gene density, with red indicating regions of high gene density and blue indicating of low gene density. (**b**) Collinearity relationship of *GmAGCs* gene family members in *G. max*.

The chromosomal distribution and gene duplication analysis of *GmAGC* genes were performed using GFF genome annotations. A total of 67 *GmAGC* genes were mapped to *G. max* chromosomes, while two genes were located on chromosome scaffolds. Except for *GmAGC1-6a* (*Glyma.U032208*) and *GmAGCAGC3-1b* (*Glyma.U032208*), the *GmAGC* genes were distributed across chromosomes 2 to 20 (Figure 2a). Chromosome 10 contained the highest density of *GmAGC* genes, with seven members, whereas chromosomes 2, 6, 14, and 18 each contained two or fewer genes (Figure 2a).

Gene duplication, a major driver of gene family expansion, was analyzed through intraspecific collinearity, including whole-genome duplication (WGD), segmental duplication, and tandem duplication. *G. max* has undergone two major WGD events, approximately 58 and 13 million years ago. A total of 54 collinear gene pairs were identified, excluding *GmAGC1-4c*, *GmAGC1-6b*, *GmAGC3-1a*, *GmAGC3-1b*, *GmPHOT2a*, *GmPHOT2b*, and *GmS6K2c* (Figure 2b). All of the remaining genes displayed linear relationships, with some genes showing collinearity with one to three other genes. All collinear gene pairs were identified as segmental/WGD duplications, indicating that these duplication events significantly contributed to the expansion of the *GmAGC* gene family in soybeans (Table 2, Figure 2b).

The Ka/Ks ratio was used to evaluate the selection pressure on the duplicated *GmAGC* genes. Ka/Ks ratios of <1, =1, and >1 indicate purifying selection, neutral evolution, and positive selection, respectively. In this study, the Ka/Ks ratios for all collinear gene pairs were less than 1, suggesting strong purifying selection during the evolutionary history of these genes (Table 2). The highest Ka/Ks ratio of 0.45 was observed for the *GmNDR1c*/*GmNDR1d* gene pair, with duplication events estimated to have occurred approximately 176.75 million years ago (Table 2, Figure 2b). The duplication events primarily occurred during the *Glycine* WGD (Ks < 0.3), and 24 events among 44 genes in the AGCVIII subfamily are predicted to have originated during the Legume WGD (1.5 < Ks < 0.3). Additionally, three duplication events in the AGCVIII subfamily were traced back to the gamma whole-genome triplication (WGT) (Ks > 1.5) (Table 2 and Figures S1 and S2). These findings suggest that the expansion of the *GmAGC* gene family resulted from three rounds of genome duplication (Figures S1 and S2).

### 2.5. Motif Finding and Gene Structure Analysis of GmAGC Genes

**Figure 3:**
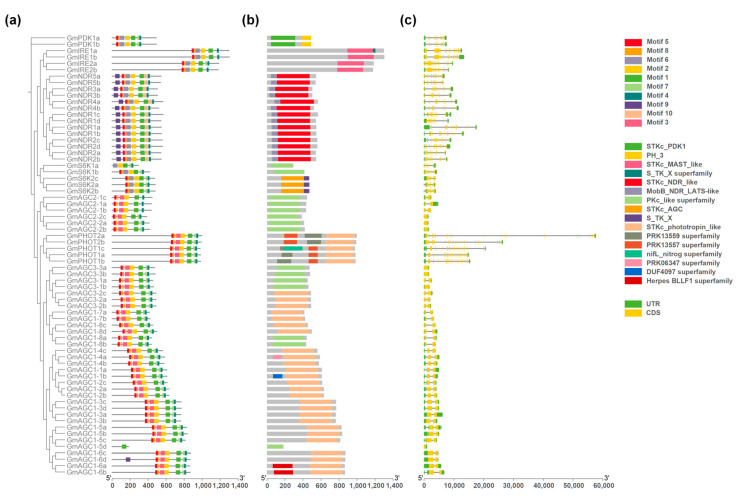
Gene structure and motif analysis of *GmAGC* genes in *Glycine max*. (**a**) Evolutionary relationship and distribution of conserved motifs of *GmAGCs* gene family members in *G. max*. (**b**) Distribution of essential domains of *GmAGCs* gene family members in *G. max*. (**c**) Gene structure of *GmAGCs* gene family members in *G. max*.

To better understand the composition and function of *GmAGC* genes, we analyzed their conserved motifs and exon–intron structures. A total of ten conserved motifs were identified, with their type, order, and number being consistent within subfamilies but differing markedly between subfamilies (Figure 3a and Table S2). In the AGCVIII subfamily, 88.64% (39 out of 44) of genes shared seven motifs (motif 1, motif 2, motif 3, motif 4, motif 5, motif 7, and motif 8) arranged in the same order. This pattern was distinct from the other four subfamilies, which lacked motif 3 (Figure 3a). Motif 6 was present in all genes across every subfamily except AGCVIII, further highlighting the differences in motif composition. Additionally, AGCVII was characterized by the presence of the unique motifs 9 and 10, which were absent in other subfamilies, indicating divergence in motif patterns. Notably, the *GmAGC1-5d* gene, which contains only motif 1, likely lost its other structural domains during diversification after gene duplication (Figure 3a). These findings suggest that, while motif composition is highly conserved within subfamilies, significant divergence exists among the five subfamilies.

The exon–intron structural analysis, performed by comparing the predicted coding sequences with their corresponding genomic sequences, provided insights into the structural evolution of *GmAGC* genes. The number of introns varied widely, ranging from 0 to 21, but genes within the same subfamily typically displayed similar exon–intron structures and consistent exon counts (Figure 3b,c and Table S3). Most genes in the AGCVI, AGCVII, and AGC (other) subfamilies contained 11 to 13 exons. In contrast, genes in the AGC1, AGC2, and AGC3 subgroups of the AGCVIII subfamily typically had only 1 to 2 exons, whereas AGC4 subfamily genes had the most and longest introns, with up to 21 (Table S3). These structural differences align with the classification of the *GmAGC* gene family and underscore the evolutionary divergence among its subfamilies.

### 2.6. Analysis of Cis-Elements in the Promoters of GmAGC Genes

**Figure 4:**
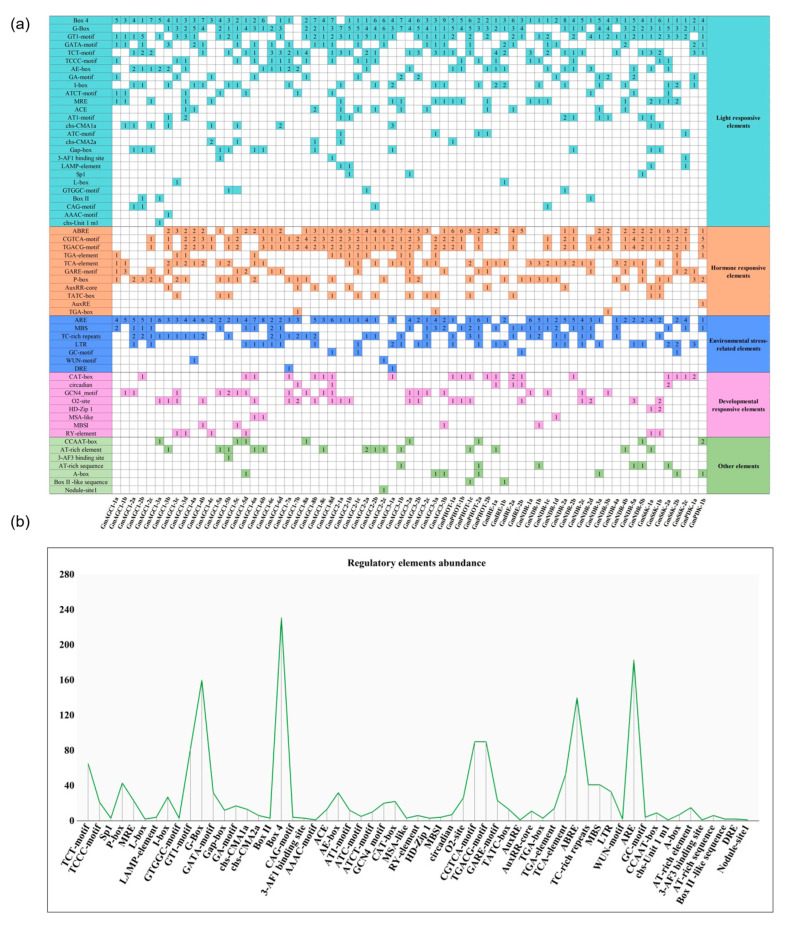
Predicted *cis*-elements of the *GmAGC* gene family: (**a**) *cis*-element analysis in the promoter region of *GmAGC* genes. The number of *cis*-elements in each gene is indicated by the number. (**b**) Frequency of *cis*-elements in the promoter regions of *GmAGCs*.

To explore the transcriptional regulation mechanisms of *GmAGC* genes, we analyzed their promoter regions, focusing on the 2000 bp upstream of the translation start sites (Figure S3). A total of 1704 *cis*-regulatory elements were identified, many of which are associated with growth, development, phytohormone signaling, light responses, and stress responses (Table S4). The most abundant element was Box4, a conserved DNA module involved in light responsiveness (Figure 4). Other frequently observed elements included the G-box (light responsiveness), ARE (anaerobic induction), ABRE (abscisic acid response), GT1-motif (salt stress response), and CGTCA-motif and TGACG-motif (MeJA response) (Figure 4). Less frequent elements included the nodule site, DRE, Box II-like sequence, chs-Unit 1 m1, 3-AF3 binding site, Box II, AAAC-motif, and TGA-box (Table S4). These results highlight the significant role of *cis*-elements in regulating *GmAGC* gene expression and offer insights into their regulatory mechanisms in *G. max*.

Salt-stress-responsive elements, including ABRE, GT1-motif, DRE/CRT, MBS (MYB binding sites), LTR (low-temperature response element), G box (bZIP transcription factor binding site), and GC-motif, were found to be widely distributed across the promoters of *GmAGC* genes (Figure 4). The presence of these elements suggests that the *GmAGC* gene family plays a significant role in soybeans’ response to salt stress, providing a foundation for understanding their function in stress adaptation and regulation.

### 2.7. Expression Patterns of GmAGC Genes in Different Tissues

To investigate the potential functions of *GmAGC* genes, we analyzed their expression profiles across various soybean tissues, including leaves, seeds, shoot apical meristems (SAMs), pods, stems, flowers, roots, root hairs, and nodules. RNA-seq data from Phytozome provided a comprehensive gene expression atlas across these tissues, enabling a detailed characterization and comparison of *GmAGC* transcript levels (Table S5). However, three genes (*GmAGC1-5d*, *GmAGC1-6b*, and *GmAGC3-1b*) lacked RNA-seq data, likely because they have not been annotated in the reference genome.

**Figure 5:**
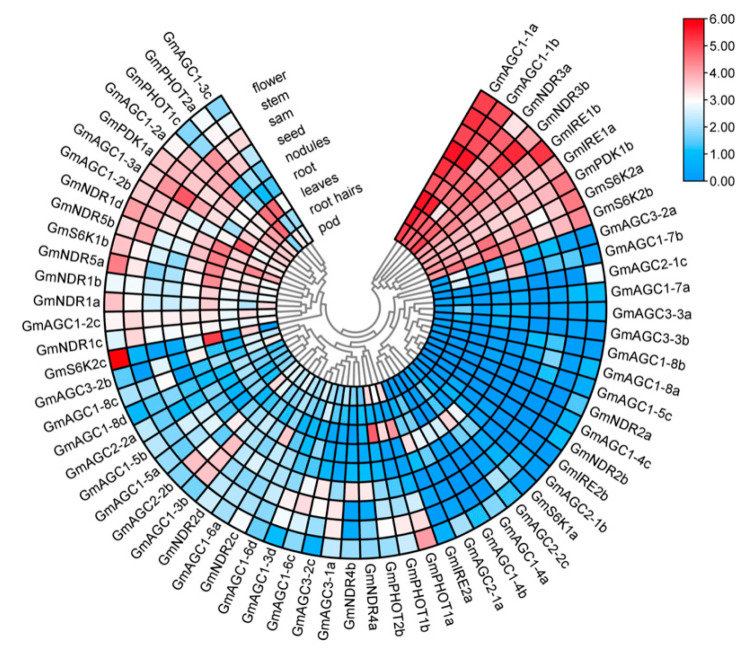
Gene expression pattern analysis of *GmAGC* in *Glycine max*.

The RNA-seq analysis identified nine *GmAGC* genes (*GmAGC1-1a*, *GmAGC1-1b*, *GmNDR3a*, *GmNDR3b*, *GmIRE1a*, *GmIRE1b*, *GmPDK1b*, *GmS6K2a*, and *GmS6K2b*) with high transcript abundance across all tissues examined (Figure 5). In contrast, several genes (*GmAGC2-1b*, *GmIRE2b*, *GmNDR2a*, *GmAGC1-5c*, *GmAGC1-8a*, and *GmAGC1-8b*) displayed low transcript levels. Most *GmAGC* genes exhibited preferential expression, with distinct peaks in one or two specific tissues (Figure 5). Interestingly, three genes (*GmNDR2a*, *GmNDR2b*, and *GmAGC1-4c*) were exclusively expressed in flowers, suggesting their specialized roles in floral development.

In soybeans, a significant proportion (87.88%) of the analyzed *GmAGC* genes were constitutively expressed across all nine tissue types, indicating that *GmAGC* genes are involved in multiple developmental processes. *GmAGC* genes were predominantly expressed in tissues associated with active cell division and development, such as roots, flowers, and SAM tissue. Among all of the analyzed tissues, approximately 19.7% (*n* = 66) of *GmAGC* genes exhibited the highest transcript levels in roots, 18.2% in flowers, 15.2% in SAM, 10.6% in stems, 10.6% in leaves, 10.6% in seeds, 7.6% in root hairs, 4.5% in nodules, and 3.0% in pods (Figure 5). Additionally, all five *GmPHOT* genes showed high transcript abundance in leaves, with *GmPHOT1c* also expressed in root hairs, which is consistent with its role as a blue light receptor.

### 2.8. Gene Expression of GmAGC Members Under Salt Stress

The presence of stress-responsive elements in the promoters of *GmAGC* genes supports previous findings that AGC kinases play a vital role in responding to abiotic stresses. To identify genes involved in the salt stress response, 54 *GmAGC* genes were selected for qRT-PCR analysis. These genes were selected from all five subfamilies of the GmAGC kinase family. Expression levels were examined in leaves at 0 h, 2 h, 6 h, and 12 h, and in roots at 0 h, 2 h, 4 h, and 6 h following the salt treatment, as roots respond faster than leaves to salt stress due to being directly immersed in the sodium chloride solution.

**Figure 6:**
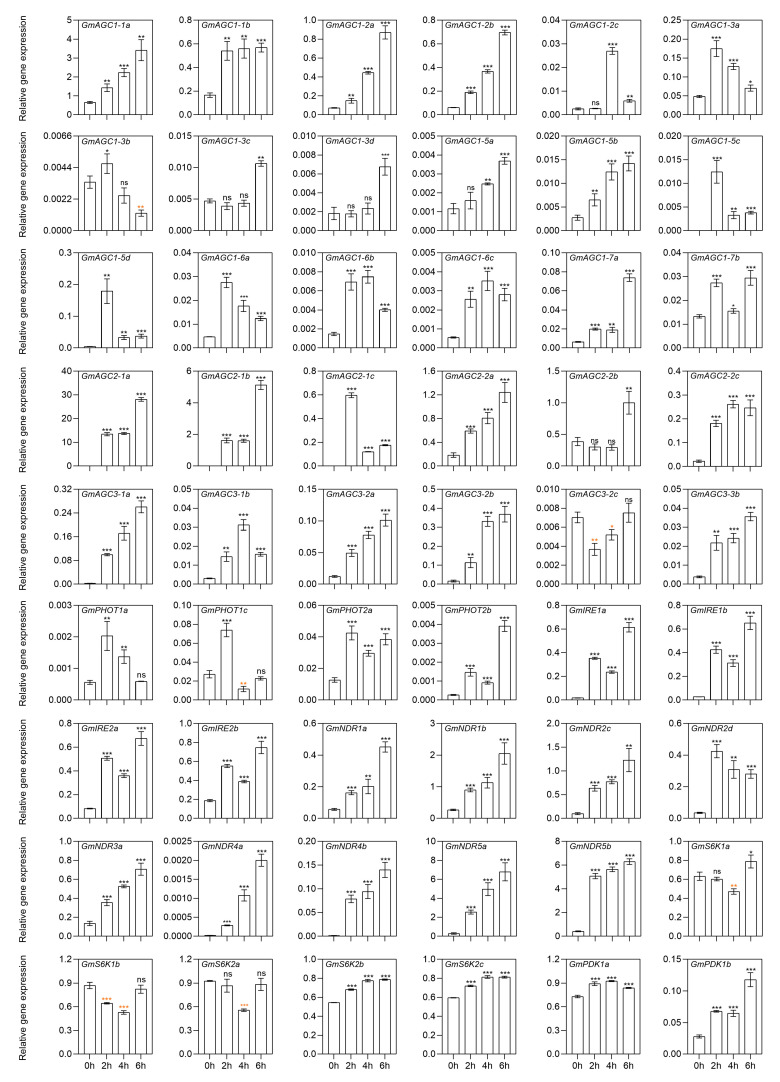
qRT-PCR expression patterns of *GmAGC* genes in roots under salt stress. The salt treatment time were 0, 2, 4, and 6 h. The black asterisks indicate significantly higher expression while red ones show significantly lower expression. * *p* < 0.05, ** *p* < 0.01, *** *p* < 0.001, ns means no significant difference, according to the Student’s *t* test.

Of the 54 genes analyzed, all were influenced by salt stress in either roots or leaves, indicating that most *GmAGC* genes are involved in the salt stress response (Figure 6 and Figure S4). The majority of these genes were upregulated in response to salt stress, though a few were downregulated. Specifically, *GmAGC3-2c*, *GmS6K1a*, *GmS6K1b*, and *GmS6K2a* were downregulated in roots, while *GmAGC1-2a*, *GmAGC1-3c*, *GmAGC1-7a*, *GmAGC1-7b*, *GmAGC2-2b*, *GmPHOT1c*, *GmPHOT2a*, *GmPHOT2b, GmIRE2b*, and *GmPDK1b* were downregulated in leaves (Figure 6 and Figure S4). In roots, several genes exhibited more than a 10-fold increase in expression after salt treatment, including *GmAGC1-2a*, *GmAGC1-2b*, *GmAGC1-2c*, *GmAGC1-5c*, *GmAGC1-5d*, *GmAGC1-7a*, *GmAGC2-1a*, *GmAGC2-1b*, *GmAGC2-1c*, *GmAGC2-2c*, *GmAGC3-1a*, *GmAGC3-1b*, *GmAGC3-2b*, *GmAGC3-3b*, *GmPHOT2b*, *GmNDR4a*, *GmNDR4b*, *GmNDR5a*, *GmNDR5b*, *GmIRE1a*, and *GmIRE1b* (Figure 6). Similarly, in leaves, genes such as *GmAGC1-1b*, *GmAGC1-2c*, *GmAGC1-3a*, *GmAGC1-5a*, *GmAGC1-5c*, *GmAGC1-5d*, *GmAGC1-6b*, *GmAGC2-1c*, *GmAGC3-1b*, *GmAGC3-3b*, *GmNDR4a*, *GmNDR4b*, *GmS6K2a*, and *GmS6K2c* exhibited more than a 10-fold increase in expression following salt treatment (Figure S4). These results suggest that upregulation is the predominant response of *GmAGC* genes under salt stress.

The genes showing increased expression in salt-treated roots can be categorized into three distinct groups. The first group includes genes that rapidly respond to salt stress, showing a notable rise in expression by 2 h, followed by a sustained or further increase in expression at 4 and 6 h. Genes such as *GmAGC1-1a*, *GmAGC2-1a*, *GmAGC3-1a*, *GmNDR1a*, *GmS6K2a*, and *GmPDK1b* all into this category. The second group includes genes with a slower response, showing no significant change in expression at 2 or 4 h and a marked upregulation only at 6 h, such as *GmAGC1-3a*, *GmAGC1-3b*, and *GmAGC2-2b*. The third group includes genes that peak at 2 or 4 h, followed by a decrease in expression, such as *GmAGC1-2c*, *GmAGC1-5c*, *GmAGC2-1c*, and *GmPHOT1a*. Similar patterns were also observed in salt-treated leaves, though some genes exhibited different expression trends between roots and leaves. While the majority of *GmAGC* genes displayed consistent expression patterns between roots and leaves, some exhibited differential or even opposite trends. For instance, *GmAGC1-2a*, *GmAGC1-7a*, *GmAGC1-7b*, *GmAGC2-2b*, *GmPHOT1c*, *GmPHOT2a*, *GmPHOT2b*, and *GmPDK1b* were upregulated in roots but downregulated in leaves. These findings suggest that specific *GmAGC* genes play distinct roles in the soybean response to salt stress. Their expression patterns reflect both spatial and temporal regulation, with each gene contributing to the plant’s overall ability to adapt to environmental challenges.

## 3. Discussion

AGC protein kinases are a subgroup of serine/threonine kinases found across eukaryotes; they play essential roles in receptor-mediated growth factor signal transduction. These kinases are critical for regulating plant growth, cell death, immunity, and responses to abiotic stresses. However, AGC protein kinases have not yet been identified in *G. max* (soybean), and the functions of *GmAGC* genes in soybeans remain largely unexplored. In this study, we identified 69 *GmAGC* genes in soybeans and conducted a comprehensive analysis of their characterization, collinearity, sequence structure, *cis*-elements, and expression patterns. Several classification systems for the AGC kinase family in plants have been proposed. In 2003, the *A. thaliana* AGC kinase family was divided into six subfamilies based on evolutionary relationships and functional differences: PDK1, AGCVI, AGCVII, AGCVIIIa (including AGC1 and AGC3), AGCVIIIb (including AGC2 and AGC4), and the AGC (other) subfamily [6]. In 2007, the AGCVIII members of *A. thaliana* were further classified into four subgroups based on their protein domains: AGC1, AGC2, AGC3, and AGC4, and the AGC kinase family was reorganized into five subfamilies: PDK1, AGCVI, AGCVII, AGCVIII, and AGC (other) [34]. This nomenclature system is widely adopted in plant research [10,40]. An alternative classification system, based on the one used for human and animal AGC kinases, divides a plant’s AGC kinase family into seven subfamilies: PDK, S6K (AGCVI), NDR (AGCVII), IRE (AGC (other)), AGC1, AGC2, and AGC2 related [5]. In this study, we classified the *GmAGC* kinase genes in soybeans into five subfamilies, PDK1, AGCVI, AGCVII, AGCVIII, and AGC (other), based on the second classical classification system and the phylogenetic relationship with *AtAGC* genes.

In contrast to the *A. thaliana* AGC family, a significantly larger number of *GmAGC* genes were identified in soybeans. This expanded gene pool likely reflects the complex evolutionary history of G. max, which has undergone one whole-genome triplication (Gamma WGT) and two whole-genome duplication events (legume WGD and *Glycine* WGD) (Figure S2) [41]. This genetic expansion is believed to have enhanced the ability of flowering plants, including soybeans, to adapt to new environments [42]. In our study, 54 gene pairs from the *GmAGC* family were found to have undergone duplication events. The earliest duplication event was observed in the *GmNDR2c*/*GmNDR1d* gene pair, which occurred during the Gamma WGT around 176.75 million years ago. Additionally, the *GmAGC2-1a*/*GmAGC2-1b* and *GmAGC2-1c*/*GmAGC2-1b* gene pairs also resulted from Gamma WGT, suggesting that these are among the more ancient *AGC* kinase genes in soybeans. Previous research has shown that *PHOT2* represents the most ancient AGCVIII kinase gene and is present in all plant species, while *PHOT1*, a duplication of the ancestral *PHOT2*, appeared later and is limited to seed plants [12]. In soybeans, no duplication was found for *GmPHOT2s* genes. Instead, the *GmPHOT1s* gene underwent duplication during both the legume WGD and *Glycine* WGD events (Figure S2). Gene replication events, including tandem duplications, fragment duplications, and transpositions, have contributed to the expansion of gene families in soybeans. The GmAGC kinase developed primarily through segmental or WGD duplication (Table 2) [43]. This pattern of gene expansion through duplication aligns with similar findings in other plant species, such as rice, where the *AGC* gene family also expanded through segmental and WGD events [16]. These findings reinforce the idea that such mechanisms are key drivers of the *AGC* gene family’s expansion in the *G. max* genome, likely contributing to the emergence of new functional genes within the *GmAGC* family. The Ka/Ks ratios for the soybean AGC kinase homologs were all less than 1, indicating that these genes have undergone purifying selection. Purifying selection eliminates harmful mutations, preserving the functional integrity of these genes over time [44]. This selective pressure suggests that AGC kinases play crucial roles in soybean physiology, and significant alterations to these genes could be detrimental to the plant. Similar patterns of purifying selection have been observed in other plant species, further confirming the conserved nature of the AGC kinase gene family across diverse plant lineages [16,18].

The gene structure of *GmAGC* genes in soybeans exhibits notable variability in terms of exon–intron organization. For instance, genes in the GmAGC1, GmAGC2, and GmAGC3 subfamilies typically contain 1 to 2 exons, whereas genes in the GmAGC4 subfamily (*GmPHOTs*) tend to have more introns, often with greater lengths (Figure 3c). The number and length of introns are closely associated with gene function, regulation, and evolutionary adaptation. Introns play a crucial role in post-transcriptional regulation, and genes with longer or more introns are more likely to undergo alternative splicing [45]. This process influences mRNA maturation timing and increases protein diversity, thereby enhancing functional complexity [46]. Such regulation allows for dynamic gene expression, especially in response to stress or environmental stimuli. Similar relationships between the intron structure and gene function have been observed in other plant species, including rice, where intron-rich genes show higher expression levels [47]. In soybean, genes with higher expression, including those in the PDK, AGCVI, AGCVII, and AGC (other) subfamilies, tend to have a higher number of introns compared to AGCVIII, which has fewer introns (Figure 3c and Table S3). Regarding protein characteristics, the GmIRE and GmAGC4 subfamilies are associated with larger proteins of higher molecular weight, suggesting more complex structural configurations linked to their biological functions. In contrast, the GmAGC and GmS6K2 subfamilies contain proteins with variable molecular weights, indicating functional diversity within these groups. This variability in protein size and structure likely reflects the diverse physiological roles of these kinases. Larger proteins may participate in more complex interactions or regulatory pathways, while smaller proteins might be involved in more direct signaling functions [48]. This functional diversification reinforces the idea that gene duplication and subsequent divergence have contributed to the specialization of gene functions within the AGC kinase family.

Gene expression patterns provide critical insights into gene function and are closely associated with the divergence of gene promoters. *Cis*-acting regulatory elements within these promoters play vital roles in regulating gene expression during development and in response to environmental stresses [49]. Among the most prevalent *cis*-elements in the promoters of *GmAGC* genes is the GT1-motif, which is involved not only in light stress but also in abiotic stress responses, particularly salinity stress [50,51]. Other salt-stress-related elements, such as ABRE, DRE/CRT, MBS, LTR, T/G box, and GC-motif, were also frequently found in these promoters [52,53,54]. The widespread presence of these stress-responsive elements emphasizes the crucial role of *GmAGC* genes in the plant’s response to abiotic stress, especially salinity. Additionally, the high occurrence of light-responsive and hormone-responsive elements in the *GmAGC* promoters, such as the G-box (light response), ABRE (abscisic acid response), and CGTCA-motif and TGACG-motif (MeJA response), suggests that these genes are tightly regulated by environmental and phytohormone signals, enabling the plant to adapt to fluctuating conditions (Figure 4 and Table S4). The involvement of *GmAGC* genes in light and hormone signaling pathways warrants further investigation to fully understand their regulatory mechanisms. Under salt stress, we observed that a notable proportion of *GmAGC* genes (50 out of 54) in roots were up-regulated in response to sodium chloride stress, indicating their involvement in the plant’s adaptive mechanisms to salt stress (Figure 6). While these findings provide valuable insights into the potential roles of AGC kinases in soybeans, limitations remain in terms of fully understanding their mechanistic functions in salt-induced stress tolerance. Further functional studies, including gene knockouts or overexpression experiments, are needed to confirm the roles of individual *GmAGC* genes in salinity stress tolerance pathways. Interestingly, while most *GmAGC* genes exhibited consistent expression patterns in salt-treated roots and leaves, some genes showed differential or even opposite expression between these tissues. This differential expression likely reflects tissue-specific adaptations to salt stress, with certain genes playing more prominent roles in one tissue over another. Such complexity in gene expression highlights the intricate regulatory networks that govern plant responses to environmental challenges [55].

## 4. Materials and Methods

### 4.1. Sequence Retrieval of AGC Protein Kinase Family Members in G. max

The *A. thaliana* Information Resource (TAIR) database (https://www.arabidopsis.org/, accessed on 7 November 2024) and the Phytozome database (https://phytozome-next.jgi.doe.gov, accessed on 7 November 2024) were used to classify the members of the *AGC* gene family. To determine the number and sequence of *AGC* kinase genes in *A. thaliana*, we retrieved the complete set of *AGC* kinase gene sequences from the TAIR database. These sequences were then used as queries for sequence comparison to identify homologous genes in soybeans (Williams 82) on the Phytozome website. The identified *AGC* kinase genes in soybeans were further validated by analyzing the presence of conserved structural domains typical of AGC kinases using the HMMER tool v3.3 (http://www.ebi.ac.uk/Tools/hmmer, accessed on 7 November 2024), ensuring the accuracy of gene family identification.

### 4.2. Phylogenetic Tree Construction, Gene Structure, and Conserved Motif Analysis of the GmAGC Gene Family

Phylogenetic analysis was performed to examine the evolutionary relationships within the *AGC* gene family of *A. thaliana* and soybean. A phylogenetic tree was constructed using protein sequences from the soybean *AGC* gene family and their corresponding homologs in *A. thaliana*. *Arabidopsis* was selected as the reference species due to its well-characterized genome and established *AGC* gene classification, which facilitated the classification of soybean *AGC* genes based on the tree. For sequence alignment, the ClustalW method was employed, and the phylogenetic tree was constructed using the neighbor-joining method in MEGA 11 with 1000 bootstrap replicates. The phylogenetic analysis results were visualized using iTOL v7 (https://itol.embl.de). Protein characteristics, including the amino acid number, molecular weight, and isoelectric point, were predicted using the Protein Parameter Calculator in TBtools-II (v2.153) [56]. The subcellular localization of the AGC proteins was analyzed using the Softberry online tool (http://www.softberry.com, accessed on 16 November 2024). A motif analysis of the protein sequences was performed using the MEME suite (https://meme-suite.org/meme/tools/meme, accessed on 16 November 2024), and the gene structure was visualized using TBtools-II.

### 4.3. Collinearity Analysis of AGC Genes

A collinearity analysis was conducted to examine *AGC* gene duplication events in the soybean genome using the MCScanX toolkit v1.0 with default parameters. This analysis identified and visualized duplicated *AGC* gene pairs, which were subsequently mapped across the soybean genome using TBtools-II. To further investigate the evolutionary dynamics of these duplicated genes, the Simple Ka/Ks tool in TBtools-II was used to calculate nonsynonymous (Ka) and synonymous (Ks) substitution rates, along with their Ka/Ks ratios. The divergence time (T) for each duplicated gene pair was estimated using the formula T = Ks/(2 × 9.1 × 10^−9^) × 10^−6^ million years ago (Mya), providing insights into the timing of gene duplication events within the soybean genome [57]. The Ks value range was assessed based on the criteria outlined by Severin et al. [41]. A Ks value greater than 1.5 corresponds to the divergence time within the whole-genome triplication (WGT) event (~130 to 240 Mya), also known as the Gamma event. A Ks value lower than 0.3 indicates divergence during the Glycine whole-genome duplication (WGD) event (~13 Mya), while Ks values between 0.3 and 1.5 correspond to the Legume WGD event (~58 Mya).

### 4.4. Cis-Acting Elements Analysis of GmAGC Genes

Promoter sequences 2 kb upstream of the transcription start sites of *GmAGC* genes in *G. max* were selected for analysis to identify *cis*-acting regulatory elements. These sequences were analyzed to identify the *cis*-acting elements involved in gene regulation. *Cis*-elements were predicted using the PlantCARE online tool (http://bioinformatics.psb.ugent.be/webtools/plantcare/html/, accessed on 22 November 2024), which offers a comprehensive database of plant regulatory elements. The cis-acting elements of *GmAGC* genes were then visualized using TBtools-II. 

### 4.5. Gene Expression Analysis of GmAGC Genes

Gene expression data for *GmAGC* genes across various tissues and developmental stages were obtained from the Phytozome database and visualized using TBtools-II. Salt stress treatments were carried out as follows: soybean seeds (Williams 82) were germinated on germination paper for three days and then transplanted into a 1/2 modified Hoagland nutrient solution (Coolaber, NS10115, Beijing, China) for 10 days under a 16 h light/8 h dark photoperiod at 25 °C. Subsequently, the plants were subjected to sodium chloride treatments at 0 mM and 150 mM. qRT-PCR was conducted to evaluate the expression levels of *GmAGC* genes in soybean leaves at 0, 2, 6, and 12 h after salt treatment and in roots at 0, 2, 4, and 6 h. The Ultrapure RNA Kit (CWBIO, Taizhou, China) was employed for RNA extraction. The PrimeScript™ RT reagent Kit with gDNA Eraser (Takara, Japan) was used for reverse transcription to synthesize cDNA. TB Green^®^ Premix Ex Taq™ II (Takara, Kyoto, Japan) was used for the quantitative real-time PCR reactions on the Roche PCR instrument. Relative expression levels were calculated using the 2^−ΔΔCT^ method, with *GmActin* as the internal control. For expression result analysis, the *t*-test was used for the significance analysis, and the level of significance was indicated with asterisks (* *p* < 0.05, ** *p* < 0.01, and *** *p* < 0.001). Primer sequences for qRT-PCR are provided in Supplementary Table S6.

## 5. Conclusions

In this study, we identified 69 *AGC* kinase genes in *G. max* (soybean), classified into five subfamilies, and examined their roles in salinity stress responses. Our findings demonstrate significant expansion of the *GmAGC* gene family through gene duplications, particularly within the AGCVI, AGCVII, and AGCVIII subfamilies. The gene expression analysis revealed that most *GmAGC* genes are upregulated in roots and leaves under salt stress, underscoring their essential role in stress adaptation. The presence of stress-responsive cis-elements in gene promoters further confirms their involvement in salinity tolerance. These findings enhance our understanding of the molecular mechanisms underlying salt stress tolerance in *G. max* and provide valuable insights for improving crop strategies against abiotic stress.

## Figures and Tables

**Table 1:** Basic information of *GmAGC* family genes and their proteins in *Glycine max*.

Sr.no.	Gene	Locus ID	Chr	Size (aa)	MW(Da)	pI	Subcellular Location	AGC Kinase Classification	
1	*GmAGC1-1a*	*Glyma.09G015400*	9	608	66,157.12	8.82	Cytoplasm	AGC1	AGCⅧ
2	*GmAGC1-1b*	*Glyma.15G121200*	15	608	66,112.15	8.51	Cytoplasm		
3	*GmAGC1-2a*	*Glyma.16G070700*	16	631	69,937.32	7.17	Plasma membrane		
4	*GmAGC1-2b*	*Glyma.19G060500*	19	631	69,931.22	7.98	Plasma membrane		
5	*GmAGC1-2c*	*Glyma.19G003800*	19	612	68,735.18	8.02	Plasma membrane		
6	*GmAGC1-3a*	*Glyma.03G111200*	3	763	83,526.82	6.20	Extracellular		
7	*GmAGC1-3b*	*Glyma.07G114400*	7	763	83,407.83	6.26	Extracellular		
8	*GmAGC1-3c*	*Glyma.09G242900*	9	766	83,736.08	6.99	Extracellular		
9	*GmAGC1-3d*	*Glyma.18G251800*	18	768	83,844.12	7.22	Extracellular		
10	*GmAGC1-4a*	*Glyma.08G160400*	8	586	64,793.02	8.89	Cytoplasm		
11	*GmAGC1-4b*	*Glyma.15G266800*	15	576	63,503.47	8.14	Cytoplasm		
12	*GmAGC1-4c*	*Glyma.08G232800*	8	560	62,559.09	9.23	Cytoplasm		
13	*GmAGC1-5a*	*Glyma.04G111700*	4	827	90,218.08	6.78	Extracellular		
14	*GmAGC1-5b*	*Glyma.06G322600*	6	835	90,986.97	6.72	Extracellular		
15	*GmAGC1-5c*	*Glyma.12G002700*	12	813	90,613.40	9.00	Extracellular		
16	*GmAGC1-5d*	*Glyma.09G234251*	9	182	19,986.92	5.76	Plasma membrane		
17	*GmAGC1-6a*	*Glyma.10G067400*	10	863	93,847.54	8.67	Extracellular		
18	*GmAGC1-6b*	*Glyma.U031420*	un	866	93,986.43	8.65	Extracellular		
19	*GmAGC1-6c*	*Glyma.03G193000*	3	871	95,373.38	8.83	Extracellular		
20	*GmAGC1-6d*	*Glyma.19G193100*	19	868	95,279.47	8.84	Extracellular		
21	*GmAGC1-7a*	*Glyma.10G203100*	10	414	45,918.41	6.40	Plasma membrane		
22	*GmAGC1-7b*	*Glyma.20G187200*	20	422	46,799.55	6.02	Plasma membrane		
23	*GmAGC1-8a*	*Glyma.11G187600*	11	441	48,991.94	6.44	Plasma membrane		
24	*GmAGC1-8b*	*Glyma.12G086900*	12	436	48,490.60	7.68	Nuclear		
25	*GmAGC1-8c*	*Glyma.12G182600*	12	453	50,636.74	6.66	Cytoplasm		
26	*GmAGC1-8d*	*Glyma.13G318400*	13	497	55,485.34	6.32	Plasma membrane		
27	*GmAGC3-1a*	*Glyma.13G220100*	13	452	50,551.26	8.29	Plasma membrane	AGC3	
28	*GmAGC3-1b*	*Glyma.U032208*	un	460	51,632.71	8.79	Plasma membrane		
29	*GmAGC3-2a*	*Glyma.05G009200*	5	488	55,368.00	9.28	Plasma membrane		
30	*GmAGC3-2b*	*Glyma.17G117300*	17	490	55,622.27	9.39	Plasma membrane		
31	*GmAGC3-2c*	*Glyma.04G148400*	4	488	54,931.86	9.09	Plasma membrane		
32	*GmAGC3-3a*	*Glyma.08G174400*	8	472	53,236.81	9.35	Plasma membrane		
33	*GmAGC3-3b*	*Glyma.15G252700*	15	478	54,469.23	9.44	Plasma membrane		
34	*GmPHOT1a*	*Glyma.13G330400*	13	982	110,192.34	7.29	Plasma membrane	AGC4	
35	*GmPHOT1b*	*Glyma.15G043600*	15	982	110,324.67	8.23	Plasma membrane		
36	*GmPHOT1c*	*Glyma.12G074100*	12	977	109,600.73	7.06	Plasma membrane		
37	*GmPHOT2a*	*Glyma.08G264900*	8	996	111,560.13	6.36	Plasma membrane		
38	*GmPHOT2b*	*Glyma.16G096600*	16	990	110,960.66	7.59	Plasma membrane		
39	*GmAGC2-1a*	*Glyma.03G088800*	3	436	49,579.15	6.62	Plasma membrane	AGC2	
40	*GmAGC2-1b*	*Glyma.16G084700*	16	434	49,826.72	7.69	Extracellular		
41	*GmAGC2-1c*	*Glyma.08G345500*	8	445	50,615.27	7.21	Extracellular		
42	*GmAGC2-2a*	*Glyma.11G131800*	11	411	46,524.23	9.53	Plasma membrane		
43	*GmAGC2-2b*	*Glyma.12G056000*	12	419	47,344.31	9.71	Plasma membrane		
44	*GmAGC2-2c*	*Glyma.13G341400*	13	386	43,528.03	9.65	Plasma membrane		
45	*GmIRE1a*	*Glyma.07G103400*	7	1297	142,803.63	5.81	Nuclear	AGC other	
46	*GmIRE1b*	*Glyma.09G174000*	9	1302	143,144.25	5.64	Nuclear		
47	*GmIRE2a*	*Glyma.09G232400*	9	1184	132,490.49	5.41	Nuclear		
48	*GmIRE2b*	*Glyma.12G004100*	12	1176	131,889.99	5.49	Nuclear		
49	*GmNDR1a*	*Glyma.02G003200*	2	547	63,207.30	7.20	Nuclear	AGCⅦ	
50	*GmNDR1b*	*Glyma.10G005600*	10	547	63,353.58	8.15	Nuclear		
51	*GmNDR1c*	*Glyma.10G181500*	10	566	66,294.51	7.85	Nuclear		
52	*GmNDR1d*	*Glyma.20G208900*	20	543	62,958.90	8.45	Nuclear		
53	*GmNDR2a*	*Glyma.03G164600*	3	542	62,867.10	5.84	Cytoplasm		
54	*GmNDR2b*	*Glyma.19G166000*	19	546	63,507.89	5.88	Nuclear		
55	*GmNDR2c*	*Glyma.10G038400*	10	558	64,301.02	6.37	Cytoplasm		
56	*GmNDR2d*	*Glyma.13G124800*	13	559	64,545.18	6.21	Cytoplasm		
57	*GmNDR3a*	*Glyma.04G053900*	4	503	58,226.48	5.80	Cytoplasm		
58	*GmNDR3b*	*Glyma.06G054200*	6	503	58,254.45	5.80	Cytoplasm		
59	*GmNDR4a*	*Glyma.09G066300*	9	563	64,847.96	6.50	Extracellular		
60	*GmNDR4b*	*Glyma.15G172300*	15	519	59,615.93	6.14	Cytoplasm		
61	*GmNDR5a*	*Glyma.14G083100*	14	544	62,311.44	6.69	Extracellular		
62	*GmNDR5b*	*Glyma.17G242000*	17	538	61,522.02	5.77	Extracellular		
63	*GmS6K1a*	*Glyma.05G032000*	5	291	32,603.31	7.69	Plasma membrane	AGCⅥ	
64	*GmS6K1b*	*Glyma.17G094700*	17	415	46,873.57	7.28	Nuclear		
65	*GmS6K2a*	*Glyma.09G273600*	9	1005	112,107.84	7.34	Plasma membrane		
66	*GmS6K2b*	*Glyma.18G212800*	18	479	54,328.16	6.21	Plasma membrane		
67	*GmS6K2c*	*Glyma.14G188100*	14	472	53,323.71	7.24	Nuclear		
68	*GmPDK1a*	*Glyma.10G200600*	10	491	54,802.36	6.73	Cytoplasm	PDK1	
69	*GmPDK1b*	*Glyma.20G190000*	20	491	54,731.24	6.73	Cytoplasm		

un, unknown.

**Table 2:** The Ka/Ks values in duplicated gene pairs in *Glycine max*.

Gene Pairs	Ka	Ks	Ka/Ks	Date (Mya)	Type of Selection	Type of Duplication
*GmAGC1-1a/GmAGC1-1b*	0.02	0.15	0.13	12.45	Purifying	Segmental/WGD
*GmAGC1-2a/GmAGC1-2b*	0.09	0.65	0.13	53.34	Purifying	Segmental/WGD
*GmAGC1-2a/GmAGC1-2c*	0.02	0.17	0.09	14.34	Purifying	Segmental/WGD
*GmAGC1-2c/GmAGC1-2b*	0.08	0.67	0.12	54.95	Purifying	Segmental/WGD
*GmAGC1-3a/GmAGC1-3b*	0.02	0.13	0.13	11.02	Purifying	Segmental/WGD
*GmAGC1-3a/GmAGC1-3c*	0.08	0.62	0.13	50.53	Purifying	Segmental/WGD
*GmAGC1-3a/GmAGC1-3d*	0.08	0.56	0.15	45.67	Purifying	Segmental/WGD
*GmAGC1-3b/GmAGC1-3c*	0.08	0.62	0.14	50.44	Purifying	Segmental/WGD
*GmAGC1-3b/GmAGC1-3d*	0.09	0.56	0.16	46.02	Purifying	Segmental/WGD
*GmAGC1-3c/GmAGC1-3d*	0.02	0.14	0.12	11.78	Purifying	Segmental/WGD
*GmAGC1-4a/GmAGC1-4b*	0.02	0.16	0.15	13.17	Purifying	Segmental/WGD
*GmAGC1-5a/GmAGC1-5b*	0.02	0.09	0.25	7.74	Purifying	Segmental/WGD
*GmAGC1-5d/GmAGC1-5c*	0.05	0.12	0.41	10.04	Purifying	Segmental/WGD
*GmAGC1-6a/GmAGC1-6d*	0.13	0.45	0.29	36.89	Purifying	Segmental/WGD
*GmAGC1-6c/GmAGC1-6a*	0.13	0.43	0.31	35.39	Purifying	Segmental/WGD
*GmAGC1-6c/GmAGC1-6d*	0.02	0.10	0.25	7.83	Purifying	Segmental/WGD
*GmAGC1-7a/GmAGC1-7b*	0.04	0.22	0.17	17.69	Purifying	Segmental/WGD
*GmAGC1-8a/GmAGC1-8b*	0.04	0.23	0.18	19.15	Purifying	Segmental/WGD
*GmAGC1-8a/GmAGC1-8c*	0.17	0.79	0.22	64.52	Purifying	Segmental/WGD
*GmAGC1-8a/GmAGC1-8d*	0.17	0.85	0.20	69.56	Purifying	Segmental/WGD
*GmAGC1-8b/GmAGC1-8c*	0.18	0.79	0.23	64.59	Purifying	Segmental/WGD
*GmAGC1-8b/GmAGC1-8d*	0.16	0.88	0.19	72.14	Purifying	Segmental/WGD
*GmAGC1-8c/GmAGC1-8d*	0.02	0.17	0.11	14.16	Purifying	Segmental/WGD
*GmAGC2-1a/GmAGC2-1b*	0.28	1.53	0.18	125.04	Purifying	Segmental/WGD
*GmAGC2-1a/GmAGC2-1c*	0.09	0.35	0.26	28.69	Purifying	Segmental/WGD
*GmAGC2-1c/GmAGC2-1b*	0.30	1.54	0.20	126.41	Purifying	Segmental/WGD
*GmAGC2-2a/GmAGC2-2b*	0.05	0.20	0.24	16.69	Purifying	Segmental/WGD
*GmAGC2-2a/GmAGC2-2c*	0.21	0.84	0.25	68.60	Purifying	Segmental/WGD
*GmAGC2-2b/GmAGC2-2c*	0.20	0.93	0.22	76.50	Purifying	Segmental/WGD
*GmAGC3-2a/GmAGC3-2b*	0.03	0.18	0.17	14.35	Purifying	Segmental/WGD
*GmAGC3-2c/GmAGC3-2a*	0.17	1.00	0.17	82.04	Purifying	Segmental/WGD
*GmAGC3-2c/GmAGC3-2b*	0.15	0.90	0.17	73.81	Purifying	Segmental/WGD
*GmAGC3-3a/GmAGC3-3b*	0.06	0.27	0.23	21.76	Purifying	Segmental/WGD
*GmIRE1a/GmIRE1b*	0.01	0.09	0.16	7.49	Purifying	Segmental/WGD
*GmIRE2a/GmIRE2b*	0.03	0.10	0.27	8.06	Purifying	Segmental/WGD
*GmNDR1a/GmNDR1b*	0.01	0.08	0.15	6.91	Purifying	Segmental/WGD
*GmNDR1a/GmNDR1c*	0.09	0.45	0.19	36.99	Purifying	Segmental/WGD
*GmNDR1c/GmNDR1d*	0.06	0.14	0.45	11.09	Purifying	Segmental/WGD
*GmNDR2a/GmNDR2b*	0.08	0.64	0.12	52.49	Purifying	Segmental/WGD
*GmNDR2a/GmNDR2c*	0.08	0.63	0.13	51.47	Purifying	Segmental/WGD
*GmNDR2a/GmNDR2d*	0.04	0.18	0.22	14.96	Purifying	Segmental/WGD
*GmNDR2c/GmNDR1d*	0.11	2.16	0.05	176.75	Purifying	Segmental/WGD
*GmNDR2c/GmNDR2b*	0.08	0.69	0.11	56.93	Purifying	Segmental/WGD
*GmNDR2c/GmNDR2d*	0.02	0.09	0.19	6.97	Purifying	Segmental/WGD
*GmNDR2d/GmNDR2b*	0.08	0.64	0.13	52.77	Purifying	Segmental/WGD
*GmNDR3a/GmNDR3b*	0.01	0.10	0.05	8.25	Purifying	Segmental/WGD
*GmNDR4a/GmNDR4b*	0.02	0.10	0.18	8.02	Purifying	Segmental/WGD
*GmNDR5a/GmNDR5b*	0.03	0.15	0.22	12.65	Purifying	Segmental/WGD
*GmPDK1a/GmPDK1b*	0.01	0.07	0.15	5.37	Purifying	Segmental/WGD
*GmPHOT1a/GmPHOT1b*	0.02	0.15	0.16	12.22	Purifying	Segmental/WGD
*GmPHOT1c/GmPHOT1a*	0.12	0.66	0.19	54.33	Purifying	Segmental/WGD
*GmPHOT1c/GmPHOT1b*	0.12	0.68	0.18	55.45	Purifying	Segmental/WGD
*GmS6K1a/GmS6K1b*	0.07	0.17	0.44	13.55	Purifying	Segmental/WGD
*GmS6K2a/GmS6K2b*	0.02	0.14	0.17	11.25	Purifying	Segmental/WGD

Ka, the ratio of noon-synonymous substitution; Ks, the ratio of synonymous substitution; Mya, million years ago.

## Data Availability

All data generated or analyzed in this study are included in the main text and its Supplementary Materials.

## Supplementary Materials

The following supporting information can be downloaded at: https://www.mdpi.com/article/10.3390/ijms26062588/s1.
