# Supplementary material for: Genome-Wide Analysis of AGC Genes Related to Salt Stress in Soybeans (Glycine max)
Source: Int J Mol Sci. 2025 Mar 13;26(6):2588. doi: 10.3390/ijms26062588 (PMC11941997; doi:10.3390/ijms26062588)
Supplement: Supplementary file 1 [file ijms-26-02588-s001.zip › Supplementary figure 1-4.pdf]

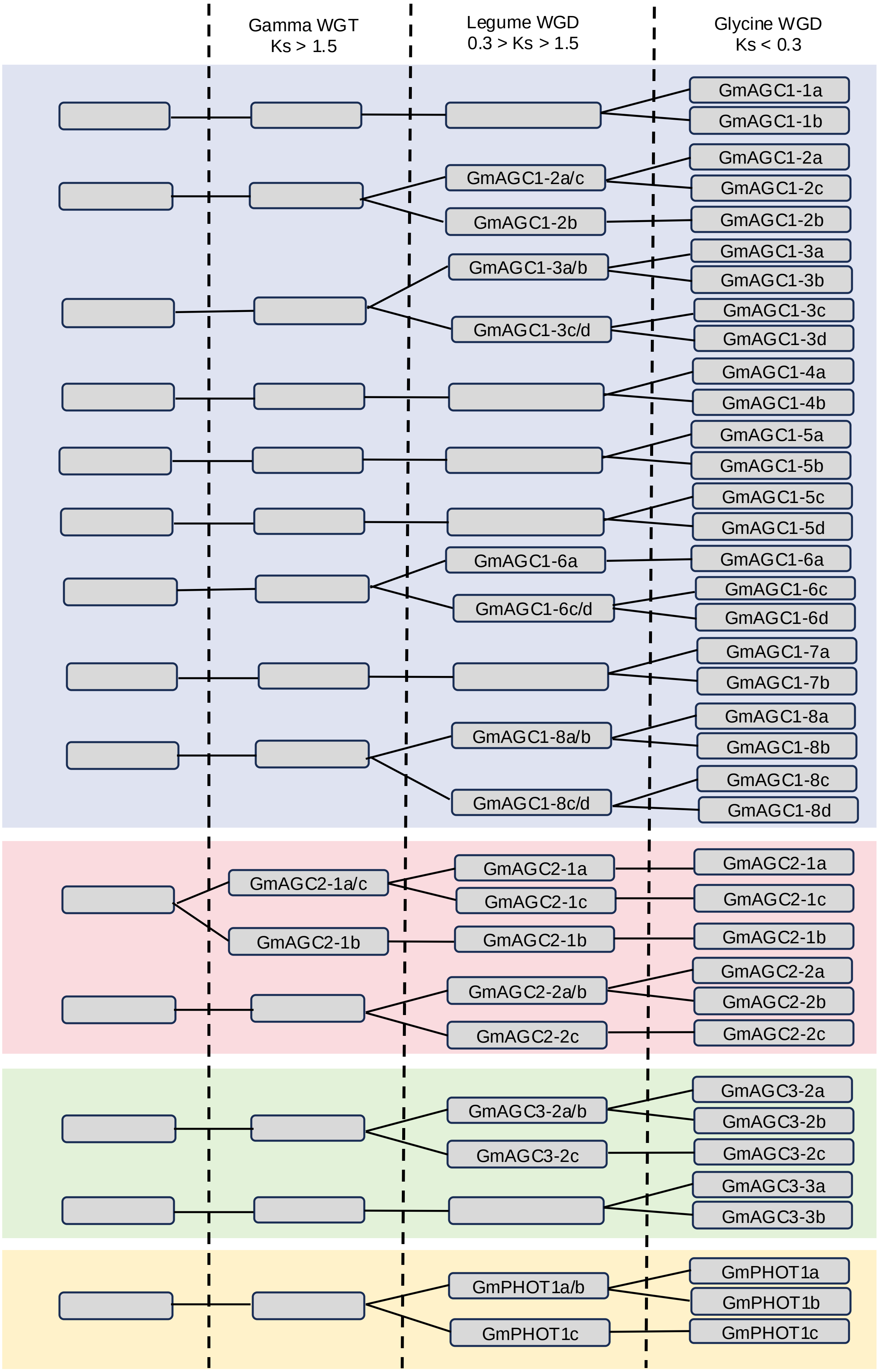

Figure S1. The deduced evolutionary models for AGCVIII subfamily genes in the evolution process of the soybean genome.
AGC1 group is represented by blue backgrounds, AGC2 group by red backgrounds, AGC3 group by green backgrounds, and AGC4 group by yellow backgrounds.

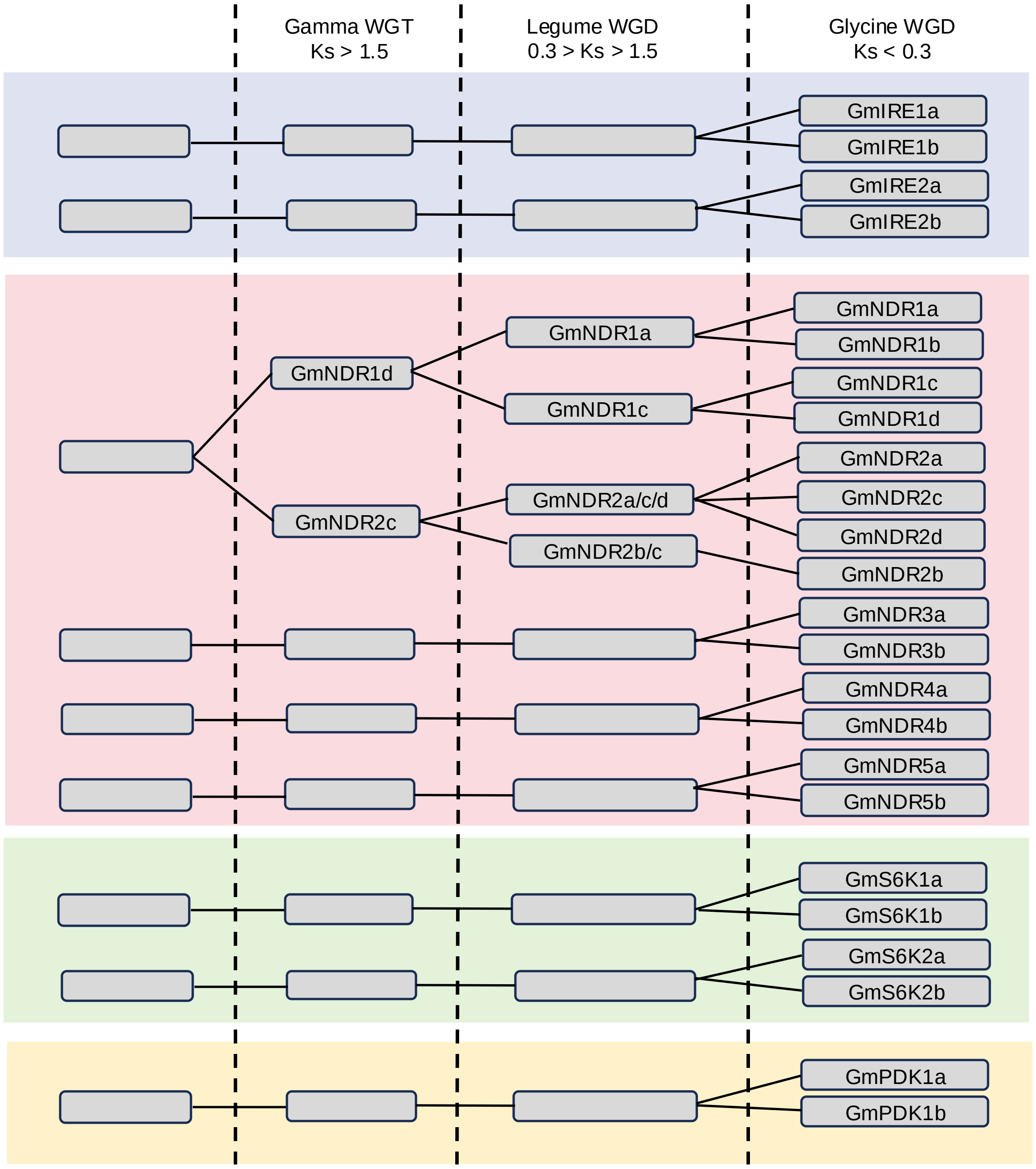

Figure S2. The deduced evolutionary models of PDK1, AGCVI, AGCVII, and AGC other subfamily genes in the evolution process of the soybean genome.
AGC other subfamily is represented by blue backgrounds, AGCVII subfamily by red backgrounds, AGCVI subfamily by green backgrounds, and PDK1 subfamily by yellow backgrounds.

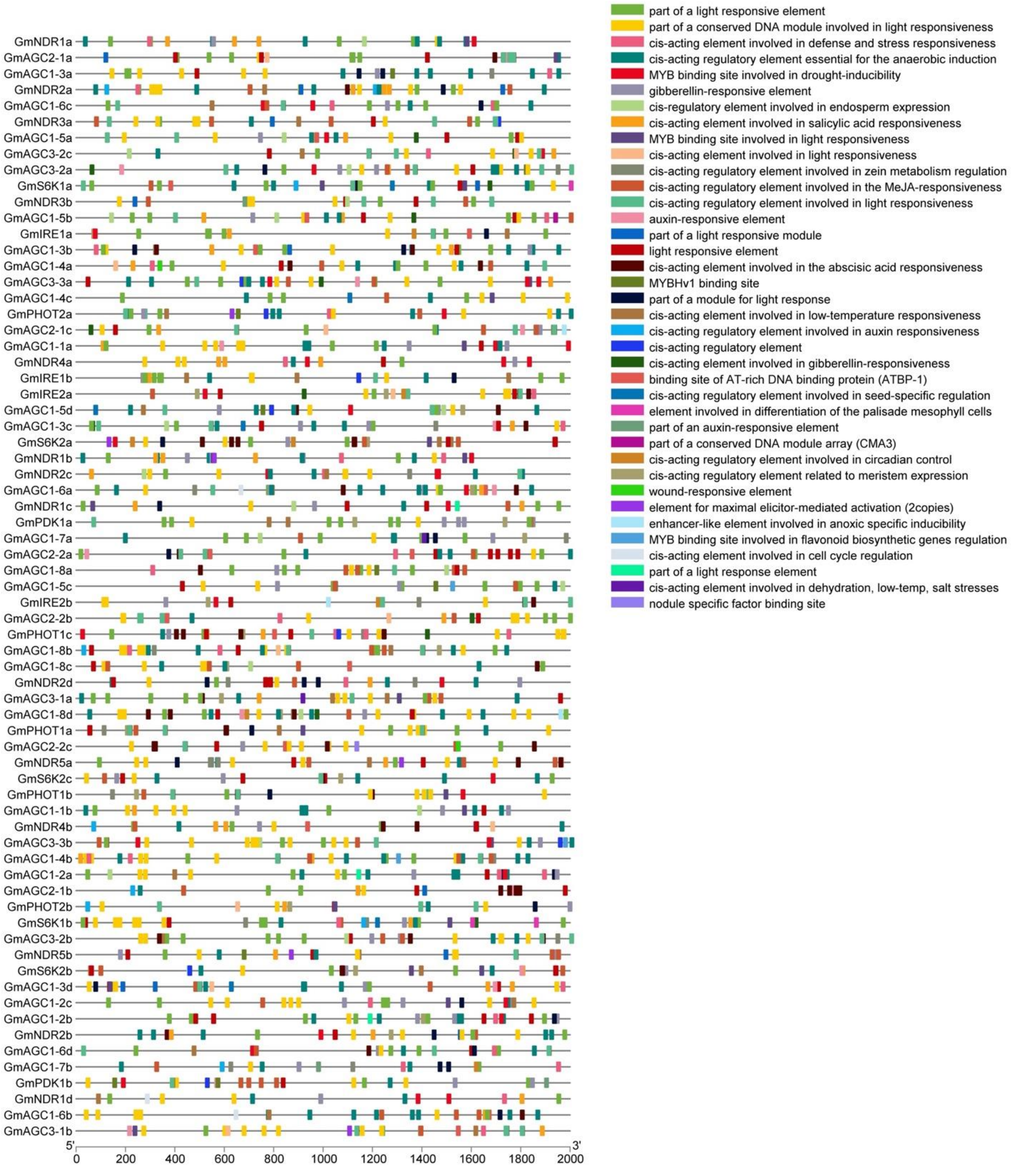

Figure S3. Predicted *c*is-element of the *GmAGC* gene family.

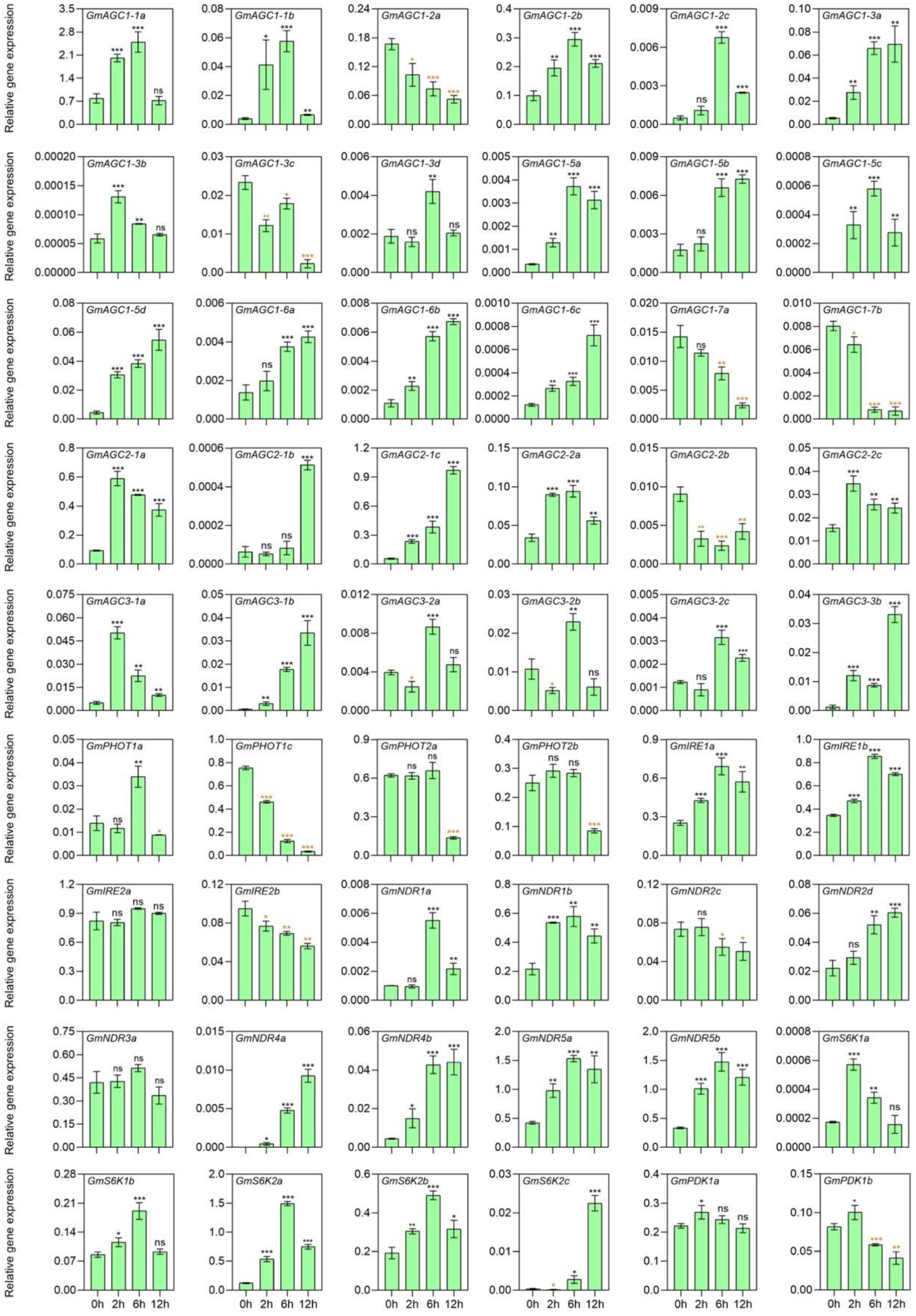

Figure S4. qRT-PCR expression patterns of *GmAGC* in leaves under salt stress.
The salt treatment time were 0, 2, 6, and 12 hours (h). The black asterisks indicate significantly higher expression while red ones show significantly lower expression. *p<0.05, **p<0.01, ***p<0.001, , ns means no significant difference, by Student's *t* test.
